# Supplementary material for: Chemophoresis engine: A general mechanism of ATPase-driven cargo transport
Source: PLoS Comput Biol. 2022 Jul 25;18(7):e1010324. doi: 10.1371/journal.pcbi.1010324 (PMC9363008; doi:10.1371/journal.pcbi.1010324)
Supplement: S1 Text — (PDF) [file pcbi.1010324.s001.pdf]

SUPPORTING INFORMATION

S1 TEXT

DETAILS OF THE DERIVATION OF THE CHEMOPHORESIS ENGINE

We first discuss the chemical thermodynamics and the corresponding statistical mechanics required for the derivation of the chemophoresis force discussed in the following paragraphs.

Rate equation at chemical equilibrium

Consider a macroscopic object, such as organelles or macromolecules that have several binding sites on their surface for chemical reactions to occur. We considered the chemical equilibrium of the reactions at a given temperature T and a chemical pool containing a chemical X with a chemical potential μ. m molecules of X in an environmental solution are attached to binding site B on the bead and form complex Y, as represented by the following reaction: $\mathrm{mX} + \mathrm{B} \underset{k_-}{\overset{k_+}{\rightleftharpoons}} \mathrm{Y}$. The binding of ligands with a receptor on the membrane is a typical example. The molecular number of the complexes (unbound sites) on the surface is denoted by $N_Y (N_B)$. Note that we defined the bead as a macroscopic entity relative to an X molecule. Under a constant site number $N_B + N_Y = N$, $\phi_{Y,B} = \frac{N_{Y,B}}{N}$ was defined as the volume fraction of Y and B on the bead, respectively. The chemical potential μ of the X molecules in the environmental solution was assumed to be constant. The rate equation of the reaction was written as follows: $\frac{d\phi_Y}{dt} = k'_+(\mu)\phi_B - k_-\phi_Y = -\frac{d\phi_B}{dt}$, where $k'_+(\mu) = k_+ \mathrm{e}^{m\beta(\mu-\bar{\mu})}$. The reaction was assumed to reach chemical equilibrium, $\frac{d\phi_Y}{dt} = -\frac{d\phi_B}{dt} = 0$. Therefore at equilibrium,

$$\phi_Y = \frac{N_Y}{N} = \frac{\mathrm{e}^{(\Delta\epsilon - m\mu)/k_BT}}{1 + \mathrm{e}^{(\Delta\epsilon - m\mu)/k_BT}}, \qquad \phi_B = \frac{N_B}{N} = \frac{1}{1 + \mathrm{e}^{(\Delta\epsilon - m\mu)/k_BT}}$$

Here $\Delta\epsilon = \bar{\mu}_Y - \bar{\mu}_B$. This result was simultaneously satisfied by $N_B + N_Y = N$ and the condition of the chemical equilibrium $m\mu = \mu_Y - \mu_B$.

Free energy changes among chemical equilibrium states attached to the chemical pool

Thermodynamics describes transitions among equilibrium states. Under isothermal and fixed surface area conditions, the change in the free energy of the bead can be represented as follows: $dF(N_Y, N_B) = \mu_Y dN_Y + \mu_B dN_B$, whereas that of the environment is $dF^{env}(x) = \mu dN_X^{env}$. Note that $-dN_B = dN_Y = -m dN_X^{env}$ from a stoichiometric relation, $\nu_X = -m, \nu_B = -1, \nu_Y = 1$ defined by $0 \rightleftharpoons \nu_{\mathrm{X}}\mathrm{X} + \nu_{\mathrm{B}}\mathrm{B} + \nu_{\mathrm{Y}}\mathrm{Y}$ related to the above reaction. Hence the change of the free energy was obtained as follows: $dF(N_Y) = (\mu_Y - \mu_B)dN_Y$. Rewriting $dF(N_Y)$ using an extent of reaction θ, $d\theta := -d\theta_B = d\theta_Y = -m d\theta_X$ and an affinity A of the bead, $A := -\sum_{i=Y,B} \nu_i \mu_i = -(\mu_Y - \mu_B) = -m\mu$. From $d\theta = d\theta_Y = dN_Y$, $dF(\theta) = -Ad\theta \quad (= m\mu d\theta)$. The affinity of the environment was $A^{env} = -\sum_{i=X} \nu_i \mu_i = m\mu$. The value of the total system was $A^{tot} = A + A^{env} = 0$.

Next we considered chemical equilibrium in a chemical pool in the environment. Using the Legendre transform for θ using $A := -(\mu_Y - \mu_B) = -m\mu$, $d\Omega(A) := dF(\theta) + d(A\theta) = \theta dA = -m\theta d\mu$. Here, $\theta = N_Y + const.$ from $d\theta = d\theta_Y = dN_Y$ (1). Therefore,

$$d\Omega(\mu) = -m N_Y d\mu \tag{S1}$$

Statistical mechanics at chemical equilibrium

Next, we considered the corresponding statistical model for the two-state system with Y (bound) and B(unbound) states for the reaction m X + B $\rightleftharpoons$ Y. The thermodynamic variables were temperature T, the affinity of the reaction A, and the extent of reaction θ, whereas microscopic variables were the binding energy ϵ and binding state n. The chemical equilibrium was sustained by a chemical bath containing a chemical X with a chemical potential μ, where the equilibrium condition is $A^{tot} = A + A^{env} = -(\mu_Y - \mu_B) + m\mu = 0$.

If the system was in the bound (unbound) state, the change in its energy was $\epsilon = \epsilon_Y(\epsilon_B)$. For a system with only one binding site, the partition function was obtained by summing the microscopic states $(\epsilon, n) = (\epsilon_Y, 1), (\epsilon_B, 0) : \Xi(T, A) := \sum_{(\epsilon,n)=(\epsilon_Y,1),(\epsilon_B,0)} \mathrm{e}^{-(\epsilon+nA)/k_BT} = \mathrm{e}^{-\epsilon_B/k_BT} + \mathrm{e}^{-(\epsilon_Y+A)/k_BT}$. Assuming that the chemical adsorption reactions at the N binding sites were independent of each other, the grand potential free energy for the system with N binding sites was written as follows:

$$\begin{aligned}\Omega(T, A) &= -Nk_BT \ln \Xi(T, A) = -Nk_BT \ln [\mathrm{e}^{-\epsilon_B/k_BT} + \mathrm{e}^{-(\epsilon_Y+A)/k_BT}] \\ &= N\epsilon_B - Nk_BT \ln [1 + \mathrm{e}^{-(\Delta\epsilon - m\mu)/k_BT}],\end{aligned} \tag{S2}$$

where $\Delta\epsilon := \epsilon_Y - \epsilon_B$. Here, we used $A = -(\mu_Y - \mu_B) = -m\mu$. Therefore, the macroscopic molecular numbers of the bound state N_Y was given by $N_Y = \theta = \langle n \rangle = \left(\frac{\partial \Omega(T,A)}{\partial A}\right)_T = -\frac{1}{m}\left(\frac{\partial \Omega(T,\mu)}{\partial \mu}\right)_T = N\frac{e^{(\Delta\epsilon - m\mu)/k_BT}}{1+e^{(\Delta\epsilon - m\mu)/k_BT}}$. Then, the number of unbound states was $N_B = N - N_Y = N/[1 + e^{(\Delta\epsilon - m\mu)/k_BT}]$. The mixing entropy was also obtained from the free energy: $S = -\left(\frac{\partial \Omega(T,A)}{\partial T}\right)_A = -k_B\left(N_Y \ln \frac{N_Y}{N} + N_B \ln \frac{N_B}{N}\right) = -k_B\left(N_Y \ln \phi_Y + N_B \ln \phi_B\right)$ (2).

Chemophoresis force

We refer to the biological elements defined above as beads with several reaction sites to which X molecules attach. The bead was placed at $\boldsymbol{r} = \boldsymbol{\xi}$ and moved in a d-dimensional space $\boldsymbol{r} \in \boldsymbol{R}^d$ $(d = 1, 2, 3)$. We considered an isothermal process that was homogeneous in a given space at a given temperature T, and a chemical pool containing the chemical X with a spatially dependent concentration $x(\boldsymbol{r})$ or, equivalently, the corresponding chemical potential $\mu(\boldsymbol{r})$. This gradient was assumed to be sustained externally. The X molecule was attached to binding site B on the bead and formed a complex Y as represented by the reaction $\mathrm{mX}(\boldsymbol{\xi}) + \mathrm{B} \rightleftarrows \mathrm{Y}$.

The molecular number of the complexes on the bead was denoted by N_Y. Note that we defined the bead as a macroscopic entity relative to an X molecule. To consider local equilibrium conditions, the bead was assumed to move sufficiently slowly so that the above reaction was at local chemical equilibrium at the position $\boldsymbol{r} = \boldsymbol{\xi}$. In other words, the time scale of the chemical reaction (τ_{chem}) was much smaller than that of the motion of the bead, $\tau_{bead} \gg \tau_{chem}$. With the assumption that a local equilibrium existed, we applied thermodynamics with spatially dependent thermodynamic variables. The change in the grand potential free energy of the bead was presented by $d\Omega(\boldsymbol{\xi}) = -mN_Y d\mu(\boldsymbol{\xi})$ from S1 Eq.

Then, we considered a virtual displacement of the bead. Under an infinitesimal displacement $\boldsymbol{\xi} \to \boldsymbol{\xi} + d\boldsymbol{\xi}$, the change in the grand potential was $d\Omega(\boldsymbol{\xi}) = -mN_Y d\mu(\boldsymbol{\xi}) = -mN_Y \boldsymbol{\nabla}\mu(\boldsymbol{\xi}) \cdot d\boldsymbol{\xi}$. In other words, the position of the bead, $\boldsymbol{\xi}$, was adopted as an effective independent variable instead of the chemical potential $\mu(\boldsymbol{\xi})$. Then, $\boldsymbol{\xi}$ was used as the work coordinate, and $-mN_Y \boldsymbol{\nabla}\mu(\boldsymbol{\xi})$ was the force exerted on the bead by the external environment, balanced by the force generated by the reservoir of chemical potential distribution $\mu(\boldsymbol{r})$, which also acts on the bead. Therefore, the reservoir-generated chemical gradient force was represented as follows:

$$\boldsymbol{F} = -\boldsymbol{\nabla}\Omega(\boldsymbol{\xi}) = mN_Y \boldsymbol{\nabla}\mu(\boldsymbol{\xi}). \tag{S3}$$

This expression was obtained as described below. Consider a quasi-static infinitesimal displacement $d\boldsymbol{\xi}$. Then, from the change in the grand potential, the maximum work done by the system on the external world through the reservoir is $d'W = N_Y \boldsymbol{\nabla}\mu(\boldsymbol{\xi}) \cdot d\boldsymbol{\xi}$. Considering that this work was done by the force exerted by the reservoir on the bead, that is, $d'W = \boldsymbol{F} \cdot d\boldsymbol{\xi}$, the force formula in S3 Eq. In other words, without an externally applied force, $\boldsymbol{\xi}$ evolved spontaneously such that $\Omega(\boldsymbol{\xi})$ monotonically decreased, that is, $d\Omega(\boldsymbol{\xi}) < 0$. When we considered an overdamped system, in which the kinetic energy of the bead was negligible, the phenomenological equation of motion was given by $0 = -\gamma\dot{\boldsymbol{\xi}} + \boldsymbol{F} = -\gamma\dot{\boldsymbol{\xi}} + mN_Y \boldsymbol{\nabla}\mu(\boldsymbol{\xi})$, and it was assumed that the friction constant resulting in dissipation was proportional to the velocity with the proportionality constant γ.

From the chemical equilibrium condition, $K_d^m := \frac{k_-}{k_+} = \frac{x(\boldsymbol{\xi})N_B}{N_Y}$, where $N_Y = N\frac{x(\boldsymbol{\xi})^m}{K_d^m + x(\boldsymbol{\xi})^m}$, $N_B = N - N_Y$. We used the chemical potential of a dilute solution, $\mu(\boldsymbol{r}) = \bar{\mu} + k_BT \ln x(\boldsymbol{r})$, where $\bar{\mu}$ is the standard chemical potential. Finally, the equation of motion was written as follows: $\gamma\dot{\boldsymbol{\xi}} = mN_Y \frac{x(\boldsymbol{\xi})^m}{K_d^m + x(\boldsymbol{\xi})^m} \boldsymbol{\nabla}\mu(\boldsymbol{\xi})$. This equation was a general expression for the motion of an element that has a number of binding sites for chemical adsorption under a gradient of chemical potential. We called this motion *chemophoresis* —similar to the nomenclature of typical 'phoresis' phenomena. Because of the chemophoresis force, the direction of motion of the bead increased the chemical potential.

In cellular environments, cargo to be modeled as the beads are always subjected to thermal fluctuations, which we included by adding thermal noise $\boldsymbol{\eta}(t)$ and replacing the equation with the Langevin equation, as

$$\gamma\frac{d\boldsymbol{\xi}}{dt} = mNk_BT\frac{u(\boldsymbol{\xi})^m}{{K_d}^m + u(\boldsymbol{\xi})^m}\frac{\boldsymbol{\nabla}u(\boldsymbol{\xi})}{u(\boldsymbol{\xi})} + \boldsymbol{\eta}(t)$$

with thermal noise $\langle \boldsymbol{\eta}(t) \rangle = 0$ and $\langle \boldsymbol{\eta}(t) \cdot \boldsymbol{\eta}(t') \rangle = 2d\gamma k_BT\delta(t - t')$, and γ is the friction constant. For the chemophoresis force to act effectively, the force must be larger than the thermal noise, as discussed in a previous report (4).

Chemophoresis engine for plasmid motion

We considered a plasmid $i (1 \le i \le M)$ placed into and moving in a d-dimensional space $\boldsymbol{r} \in \boldsymbol{R}^d (d = 1 \text{ or } 2)$ (Fig 1A in the main text). ParA-ATP dimers were bound to a PC on plasmid i at position $\boldsymbol{r} = \boldsymbol{\xi}_i$. m ParA-ATP dimer molecules interacted with ParB, which stimulated ParA ATPase activity at a catalytic rate k; N ParB molecules were assumed to be recruited to each PC

at $\boldsymbol{r} = \boldsymbol{\xi}_i$. Because ParA could not bind to PC when it was not combined with ATP, free ParA products were released from the PC immediately after ATP hydrolysis. Thus the reaction was written as follows:

$$\mathrm{m\,(ParA-ATP)_2 + PC_i} \underset{k_-}{\overset{k_+}{\rightleftharpoons}} \mathrm{(ParA-ATP)_{2m} - PC_i} \xrightarrow{k} \mathrm{2mParA + 2mADP + PC_i}$$

Through this reaction on the PCi at $\boldsymbol{r} = \boldsymbol{\xi}_i$, each plasmid acted as a sink for ParA-ATP and induced a concentration gradient of this protein. Here, we assumed an adiabatic approximation (chemical equilibrium) of the reaction $\mathrm{m\,(ParA-ATP)_2 + PC_i} \rightleftarrows \mathrm{(ParA-ATP)}_{2m} - \mathrm{PC_i}$. Denoting the concentration (chemical potential) of ParA-ATP dimers on a nucleoid as $u(\boldsymbol{r})$, the time evolution for the ParA-ATP concentration was generally written as follows: $\partial_t u(\boldsymbol{r}) = -\boldsymbol{\nabla}\cdot\boldsymbol{J}(\boldsymbol{r}) + \mathcal{R}(\boldsymbol{r})$. Here, $\boldsymbol{J}(\boldsymbol{r})$ is the diffusion flux defined by the Fick law for the chemical potential, $\boldsymbol{J}(\boldsymbol{r}) := -\mathcal{M}(u(\boldsymbol{r}))\boldsymbol{\nabla}\mu(\boldsymbol{r}) = D_u u(\boldsymbol{r})\boldsymbol{\nabla}\left(\frac{\delta F^{env}}{\delta u}\right)$, $\mathcal{R}(\boldsymbol{r})$ is the reaction term, and $F^{env}[\{u(\boldsymbol{r})\}]$ is the total free energy of the environment. Considering the free energy of a dilute solution, $F^{env}[\{u(\boldsymbol{r})\}] := \int d\boldsymbol{r}[\bar{\mu}u(\boldsymbol{r}) + k_B T u(\boldsymbol{r})(\ln u(\boldsymbol{r}) - 1)]$ (3). $\mathcal{R}(\boldsymbol{r}) := a\,(u_0 - u\,(\boldsymbol{r})) - kN\frac{u(\boldsymbol{r})^m}{{K_d}^m + u(\boldsymbol{r})^m}\sum_{i=1}^M \delta\left(\boldsymbol{r} - \boldsymbol{\xi}_i\right)$. Therefore, the RD equation for the ParA-ATP concentration was given as follows: $\partial_t u(\boldsymbol{r}) = D_u\boldsymbol{\nabla}^2 u\,(\boldsymbol{r}) + a\,(u_0 - u\,(\boldsymbol{r})) - kN\frac{u(\boldsymbol{r})^m}{{K_d}^m + u(\boldsymbol{r})^m}\sum_{i=1}^M \delta\left(\boldsymbol{r} - \boldsymbol{\xi}_i\right)$, where the first and second terms represent the diffusion of ParA-ATP and its chemical exchange at a constant rate a with the cytoplasmic reservoir (denoted by its concentration u_0), respectively. The last term denotes the inhibition by ParB on M PCs. $K_d = \frac{k_-}{k_+}$ is the dissociation constant, m is the Hill coefficient, and $\delta(\boldsymbol{r})$ is a delta function representing the hydrolysis reaction point. Without the last term (if $k = 0$), $u(\boldsymbol{r})$ reaches a homogenous equilibrium state, $u(\boldsymbol{r}) = u_0$. In contrast, the equations of motion for plasmids were given as $\gamma\dot{\boldsymbol{\xi}}_i = mNk_B T\frac{u(\boldsymbol{\xi}_i)^m}{{K_d}^m + u(\boldsymbol{\xi}_i)^m}\frac{\boldsymbol{\nabla}u(\boldsymbol{\xi}_i)}{u(\boldsymbol{\xi}_i)} + \boldsymbol{\eta}_i\,(t)\ (1 \le i \le M)$ with thermal noise $\langle\boldsymbol{\eta}_i(t)\rangle = 0$ and $\langle\boldsymbol{\eta}_i(t)\cdot\boldsymbol{\eta}_j(t')\rangle = 2d\gamma k_B T\delta_{ij}\delta(t - t')$, where γ is the friction constant. In these two equations, the size of the plasmid was assumed to be zero (4). To better describe the spatiotemporal profiles of ParA-ATP concentration and directed movement of plasmids, we considered plasmids as a sphere with a radius of l_b, and rewrote these equations as $\partial_t u(\boldsymbol{r}) = D_u\boldsymbol{\nabla}^2 u\,(\boldsymbol{r}) + a\,(u_0 - u(\boldsymbol{r})) - k\frac{N}{V}\frac{u(\boldsymbol{r})^m}{{K_d}^m + u(\boldsymbol{r})^m}\sum_{i=1}^M \theta\left(l_b - \left|\boldsymbol{r} - \boldsymbol{\xi}_i\right|\right)$, where V is the spherical volume of each PC, and $V = 2l_b$ for $d = 1$ and $V = \pi l_b^2$ for $d = 2$. $\theta(r)$ is a step function representing the space each PC occupies to describe the hydrolysis reaction space. Only within $\left|\boldsymbol{r} - \boldsymbol{\xi}_i\right| < l_b$, the reaction occurred. When $l_b \to 0$, $\theta(r)/V \to \delta\,(r)$, this equation was reduced to the previous model. By using dimensionless variables, $\tilde{\boldsymbol{r}} = \boldsymbol{r}/l, \tilde{t} = t/\tau, \tilde{u} = u/u_0, \tilde{l_b} = l_b/l, \tilde{K_d} = K_d/u_0, \tilde{k} = k/\tau^{-1}, \tilde{V} = V/l^d, \tilde{\boldsymbol{\xi}_i} = \boldsymbol{\xi}_i/l$, and redefining $\tilde{\boldsymbol{r}} \to \boldsymbol{r}, \tilde{t} \to t, \tilde{u} \to u, \tilde{l_b} \to l_b, \tilde{K_d} \to K_d, \tilde{k} \to k, \tilde{V} \to V, \tilde{\boldsymbol{\xi}_i} \to \boldsymbol{\xi}_i$, the normalized equation was written as follows:

$$\frac{\partial u(\boldsymbol{r})}{\partial t} = \boldsymbol{\nabla}^2 u\,(\boldsymbol{r}) + 1 - u(\boldsymbol{r}) - \chi\frac{u(\boldsymbol{r})^m}{{K_d}^m + u(\boldsymbol{r})^m}\sum_{i=1}^{M}\theta\left(l_b - \left|\boldsymbol{r} - \boldsymbol{\xi}_i\right|\right) \tag{S4}$$

$$\frac{d\boldsymbol{\xi}_i}{dt} = \varepsilon\int d\boldsymbol{r}\frac{u(\boldsymbol{\xi}_i)^m}{{K_d}^m + u(\boldsymbol{\xi}_i)^m}\frac{\boldsymbol{\nabla}u(\boldsymbol{\xi}_i)}{u(\boldsymbol{\xi}_i)}\theta\left(l_b - \left|\boldsymbol{r} - \boldsymbol{\xi}_i\right|\right) + \boldsymbol{\eta}_i\,(t) \qquad (1 \le i \le M) \tag{S5}$$

with thermal noise $\langle\boldsymbol{\eta}_i(t)\rangle = 0$ and $\langle\boldsymbol{\eta}_i(t)\cdot\boldsymbol{\eta}_j(t')\rangle = 2d\mathcal{D}\delta_{ij}\delta(t - t')$. Here, $\tau = a^{-1}$, $l^2 = D_u\tau, D_\xi = k_B T/\gamma$. $\mathcal{D} = D_\xi/D_u$ is the relative diffusion coefficient of the plasmid to that of ParA-ATP, and $\varepsilon = \mathcal{D}N/V$. $\chi = kN/V$ is the maximum rate of ParA-ATP hydrolysis by ParB on each PC.

REFERENCES

1. $const. = 0$ from the initial condition $(\theta_Y, N_Y) = (0, 0)$.
2. We used $\phi_Y + \phi_B = \mathrm{e}^{-(\bar{\mu}_Y - \mu_Y)/k_B T} + \mathrm{e}^{-(\bar{\mu}_B - \mu_B)/k_B T} = 1$ in the calculation.
3. If we studied the effect of phase separation, we could introduce F^{env} leading to the Cahn-Hilliard equation.
4. Sugawara T, Kaneko K. Chemophoresis as a driving force for intracellular organization: Theory and application to plasmid partitioning. BIOPHYSICS. 2011;7:77–88.
