# Supplementary material for: Chemophoresis engine: A general mechanism of ATPase-driven cargo transport
Source: PLoS Comput Biol. 2022 Jul 25;18(7):e1010324. doi: 10.1371/journal.pcbi.1010324 (PMC9363008; doi:10.1371/journal.pcbi.1010324)
Supplement: S2 Text — (PDF) [file pcbi.1010324.s002.pdf]

SUPPORTING INFORMATION

S2 TEXT

Simplified Equations of Chemophoresis Engine for Analytical Examination

In order to examine analytically the change of steady solutions of the plasmid dynamics against the values of $\chi = kN/(2l_b)$ and $\varepsilon = \mathcal{D}N/(2l_b)$, we simplify Eqs 3 and 4 in the main text with $M = 1$, without thermal fluctuation, and for a one-dimensional case under a periodic boundary condition. On the assumption of $u(x) \gg K_d$ over $x \in [0, L]$ resulting in $u(x)^m/(K_d{}^m + u(x)^m) \longrightarrow 1$, Eqs 3 and 4 are given as:

$$\frac{\partial u(x,t)}{\partial t} = \nabla^2 u(x,t) + 1 - u\,(x,t) - \chi\theta\,(l_b - |x - \xi(t)|) \tag{S1}$$

$$\frac{d\xi}{dt} = \varepsilon \int_{\xi - l_b}^{\xi + l_b} dx \nabla \ln u(x,t) = \varepsilon\,[\ln u(\xi + l_b, t) - \ln u(\xi - l_b, t)] \tag{S2}$$

S2 Eq gives a well-defined representation for the non-equilibrium condition under chemophoresis force on the plasmid through the (normalized) chemical potential difference from end to end $\Delta\mu := \ln u(\xi + l_b) - \ln u(\xi - l_b)$, which is self-generated by the plasmid.

By introducing a spacetime coordinate $z := x - vt$, where v is a constant velocity of the frame, and defining $u\,(x,t) := U(x - vt, t)$, S1 and S2 Eqs are rewritten as

$$U_t\,(z,t) = U_{zz}\,(z,t) + vU_z\,(z,t) + 1 - U\,(z,t) - \chi\theta\left(l_b - \left|z - z_\xi(t)\right|\right), \tag{S3}$$

$$\frac{dz_\xi}{dt} + v = \varepsilon \ln \frac{U(z_\xi + l_b)}{U(z_\xi - l_b)}, \tag{S4}$$

where U_z shows a partial differential for z and $z_\xi(t) = \xi\,(t) - vt$ is a spacetime position of the plasmid. By considering a steady state of the plasmid's position in z space, the coordinate represents a co-moving frame, where v corresponds to a steady velocity of the plasmid. A steady state equation of S3 and S4 Eqs is given from $U_t = 0$ and $\dot{z}_\xi = 0$ as:

$$0 = U_{zz}\,(z) + vU_z(z) + 1 - U(z) - \chi\theta\left(l_b - \left|z - z_\xi\right|\right) \tag{S5}$$

$$v = \varepsilon \ln \frac{U(z_\xi + l_b)}{U(z_\xi - l_b)}, \tag{S6}$$

Here, at the steady state, $z_\xi = 0$ can be set without losing generality. By solving S5 and S6 Eqs, the steady state solution $U^{st}\,(z)$ and v is obtained as:

$$U^{st}\,(z\,; v, \chi) = \begin{cases} A\,e^{\omega_+ z} + Be^{\omega_- z} + 1 & z < -l_b \\ Ce^{\omega_+ z} + De^{\omega_- z} + 1 - \chi & |z| < l_b \\ Ee^{\omega_+ z} + Fe^{\omega_- z} + 1 & l_b < z \end{cases} \tag{S7}$$

$$\begin{aligned} A = -2\frac{\chi}{\omega_+}\frac{\sinh(\omega_+ l_b)}{\omega_+ - \omega_-}\frac{1}{1 - e^{-\omega_+ L}}, B = -2\frac{\chi}{\omega_-}\frac{\sinh(\omega_- l_b)}{\omega_+ - \omega_-}\frac{1}{e^{-\omega_- L} - 1}, \\ C = \frac{\chi}{\omega_+}\frac{1}{\omega_+ - \omega_-}\frac{\sinh\omega_+(L/2 - l_b)}{\sinh(\omega_+ L/2)}, D = -\frac{\chi}{\omega_-}\frac{1}{\omega_+ - \omega_-}\frac{\sinh\omega_-(L/2 - l_b)}{\sinh(\omega_- L/2)}, \\ E = -2\frac{\chi}{\omega_+}\frac{\sinh(\omega_+ l_b)}{\omega_+ - \omega_-}\frac{1}{e^{\omega_+ L} - 1}, F = -2\frac{\chi}{\omega_-}\frac{\sinh(\omega_- l_b)}{\omega_+ - \omega_-}\frac{1}{1 - e^{\omega_- L}} \end{aligned} \tag{S8}$$

and $\omega_\pm = \frac{1}{2}\left(-v \pm \sqrt{4 + v^2}\right)$. Finally by calculating v from the following equation numerically, we obtain a relationship between v and χ (Fig 2B, green solid line in the main text):

$$v = \varepsilon \ln \frac{U^{st}\,(+l_b; v, \chi)}{U^{st}\,(-l_b; v, \chi)} = \varepsilon\Delta\mu(v, \chi) \tag{S9}$$

S9 Eq gives a self-consistent equation for v; The chemical potential difference $\Delta\mu(v, \chi)$ determined by the bead's velocity v generates the chemophoresis force acting on itself, resulting in its steady velocity v. S9 Eq for v shows a pitch-fork bifurcation at $\chi = \chi_c \sim 3.1$, and has three solutions at $\chi > \chi_c$ (S4 Fig).

Linear Stability Analysis of "Plasmid Surfing on Traveling Wave" Solution

We performed linear stability analysis of surfing-on-wave solution for S3 and S4 Eqs against external disturbances, in order to examine if tiny perturbation around the stationary solution is amplified or not. We take $U(z,\ t) = U^{st}\ (z) + e^{\lambda t}\delta U(z)$, and $z_{\xi}(t) = z_{\xi}^{st} + e^{\lambda t}\delta z_{\xi}$ and compute eigenvalues λ as a function of χ (S5 Fig). Substituting the perturbation into S3 and S4 Eqs, we obtain by using the steady solution S5 and S6 Eqs with $z_{\xi}^{st} = \dot{z}_{\xi}^{st} = 0$:

$$\lambda\ \delta U(z) = \delta U_{zz}(z) + v\ \delta U_z(z) - \delta U(z) \mp \chi\ \delta\left(l_b \mp z\right)\delta z_{\xi} \qquad (z \gtrless 0) \tag{S10}$$

$$\frac{\lambda}{\varepsilon}\delta z_{\xi} = \left[\frac{U_z^{st}\ (+l_b)}{U^{st}\ \ (+l_b)} - \frac{U_z^{st}\ (-l_b)}{U^{st}\ \ (-l_b)}\right]\delta z_{\xi} + \frac{\delta U\ (+l_b)}{U^{st}\ (+l_b)} - \frac{\delta U\ (-l_b)}{U^{st}\ (-l_b)} \tag{S11}$$

Furthermore, by setting a jump condition (1), we obtain equations:

$$\delta U_z(z)\Big|_{\pm l_b - 0}^{\pm l_b + 0} + v\ \delta U(z)\Big|_{\pm l_b - 0}^{\pm l_b + 0} - (1 + \lambda)\int_{\pm l_b - 0}^{\pm l_b + 0} dz\ \delta U(z) = \pm\chi\ \delta z_{\xi} \tag{S12}$$

$$\therefore \quad \delta U_z(\pm l_b + 0) - \delta U_z(\pm l_b - 0) = \pm\chi\delta z_{\xi} \tag{S13}$$

By solving S10 Eq with the jump condition S13 Eq, the two boundary conditions $\delta U(-L/2) = \delta U(L/2), \delta U_z(-L/2) = \delta U_z(L/2)$, and the two continuity conditions of $\delta U(z)$ at $z = \pm l_b$, $\delta U(\pm l_b - 0) = \delta U(\pm l_b + 0)$, the solution $\delta U(z)$ is obtained as a function of δz_{ξ}:

$$\delta U\left(z,\ \delta z_{\xi};\ \lambda,\ v, \chi\right) = \begin{cases} A' \exp(\kappa_+ z) + B' \exp(\kappa_- z) & z < -l_b \\ C' \exp(\kappa_+ z) + D' \exp(\kappa_- z) & |z| < l_b \\ E' \exp(\kappa_+ z) + F' \exp(\kappa_- z) & l_b < z \end{cases} \tag{S14}$$

$$\begin{aligned} A' &= \frac{2\chi\ \delta z_{\xi}}{\kappa_+ - \kappa_-}\frac{\sinh(\kappa_+ l_b)}{1 - \exp(-\kappa_+ L)}, B' = \frac{2\chi\ \delta z_{\xi}}{\kappa_+ - \kappa_-}\frac{\sinh(\kappa_- l_b)}{\exp(-\kappa_- L) - 1}, \\ C' &= -\frac{\chi\ \delta z_{\xi}}{\kappa_+ - \kappa_-}\frac{\sinh\kappa_+\left(\frac{L}{2} - l_b\right)}{\sinh\left(\kappa_+\frac{L}{2}\right)}, D' = \frac{\chi\ \delta z_{\xi}}{\kappa_+ - \kappa_-}\frac{\sinh\kappa_-\left(\frac{L}{2} - l_b\right)}{\sinh\left(\kappa_-\frac{L}{2}\right)}, \\ E' &= \frac{2\chi\ \delta z_{\xi}}{\kappa_+ - \kappa_-}\frac{\sinh(\kappa_+ l_b)}{\exp\left(\kappa_+\frac{L}{2}\right) - 1}, F' = \frac{2\chi\ \delta z_{\xi}}{\kappa_+ - \kappa_-}\frac{\sinh(\kappa_- l_b)}{1 - \exp\left(\kappa_-\frac{L}{2}\right)}, \end{aligned} \tag{S15}$$

and $\kappa_{\pm} = \frac{1}{2}\left(-v \pm \sqrt{4(1+\lambda) + v^2}\right)$. From S11 and S14 Eqs, we get an eigenvalue equation for λ:

$$0 = \frac{U_z^{st}(+l_b)}{U^{st}\ (+l_b)} - \frac{U_z^{st}(-l_b)}{U^{st}(-l_b)} + \frac{\alpha_+(\lambda, v)}{U^{st}(+l_b)}\chi - \frac{\alpha_-(\lambda, v)}{U^{st}(-l_b)}\chi - \frac{\lambda}{\varepsilon} \tag{S16}$$

Here, $U^{st}(\pm l_b)$, $U_z^{st}(\pm l_b)$, and $\alpha_{\pm}(\lambda, v)$ are given as:

$$U^{st}(\pm l_b) = 1 - \frac{\chi}{\omega_+ - \omega_-}\left[\frac{e^{\mp\omega_+\left(\frac{L}{2} - l_b\right)}}{\omega_+}\frac{\sinh\omega_+ l_b}{\sinh\omega_+\frac{L}{2}} - \frac{e^{\mp\omega_-\left(\frac{L}{2} - l_b\right)}}{\omega_-}\frac{\sinh\omega_- l_b}{\sinh\omega_-\frac{L}{2}}\right] \tag{S17}$$

$$U_z^{st}(\pm l_b) = -\frac{\chi}{\omega_+ - \omega_-}\left[e^{\mp\omega_+\left(\frac{L}{2} - l_b\right)}\frac{\sinh\omega_+ l_b}{\sinh\omega_+\frac{L}{2}} - e^{\mp\omega_-\left(\frac{L}{2} - l_b\right)}\frac{\sinh\omega_- l_b}{\sinh\omega_-\frac{L}{2}}\right] \tag{S18}$$

$$\alpha_{\pm}(\lambda, v) = \frac{1}{\kappa_+ - \kappa_-}\left[e^{\mp\kappa_+\left(\frac{L}{2} - l_b\right)}\frac{\sinh\kappa_+ l_b}{\sinh\kappa_+\frac{L}{2}} - e^{\mp\kappa_-\left(\frac{L}{2} - l_b\right)}\frac{\sinh\kappa_- l_b}{\sinh\kappa_-\frac{L}{2}}\right], \tag{S19}$$

where λ satisfying with S16 Eq are complex eigenvalues in general. Then, the stability of the plasmid-surfing solution (Fig 2C, green in the main text) and the instability of the plasmid-localized solution (Fig 2C, purple in the main text) are given by the absence of $\max \mathrm{Re}[\lambda(v)] > 0$ for $v \neq 0$ and the existence of $\max \mathrm{Re}[\lambda(0)] > 0$, respectively (S5 Fig). If S16 Eq with $v \neq 0$ contained any positive real parameter $\mathrm{Re}[\lambda(v)] > 0$, the perturbation around the traveling wave solution would be amplified to collapse. However, for χ larger than the bifurcation point $\chi_c \sim 3.1$, $\max \mathrm{Re}[\lambda(v)] = 0$ for $v \neq 0$ (S5 Fig). Here, $\mathrm{Re}[\lambda(v)] = 0$

for the surfing solution (Fig 2C, green in the main text) corresponds to Goldstone mode in physics, where the perturbation is neither amplified nor decayed, but only shifts the solution along the z axis due to the translational invariance (See (2) for details of the Goldstone mode studied mathematically in reaction-diffusion systems). Hence, the traveling wave itself is stable.

On the other hand, the localized solution is destabilized at $\chi = \chi_c$ where Re $[\lambda(0)]$ turns to be positive, whereas it regains stability at χ larger than $\chi \sim 5.9$ (S5 Fig). However, such localized solution cannot be numerically realized for $\chi > 5.5$ because $U^{st}(0)$ is negative and the solution is unphysical at $\chi \sim 5.5$ (S5 Fig, inset), where the approximation $u(x) \gg K_d$ is not valid; Therefore, a stable localized solution does not exist for $\chi \gtrsim 5.5$.

Finally, these results show that plasmid surfing on the traveling wave emerges through a symmetry-breaking transition at a critical reaction rate of ParA-ATP hydrolysis (= χ) as a pitch-fork bifurcation in dynamical systems theory (Fig 2B in the main text and S5 Fig).

Simplest case with large system size limit $L \to \infty$ and vanishing plasmid size limit $l_b \to 0$

In the limit of the large system size $L \to \infty$, where a (periodic) boundary effect can be negligible, S7 Eq is a simplified as:

$$U^{st}(z\,;v,\chi) = \begin{cases} -2\frac{\chi}{\omega_+}\frac{\sinh\omega_+ l_b}{\omega_+-\omega_-}e^{\omega_+ z}+1 & z < -l_b \\ \frac{\chi}{\omega_+-\omega_-}\left[\frac{1}{\omega_+}e^{\omega_+(z-l_b)}-\frac{1}{\omega_-}e^{\omega_-(z+l_b)}\right]+1-\chi & |z| < l_b \\ -2\frac{\chi}{\omega_-}\frac{\sinh\omega_- l_b}{\omega_+-\omega_-}e^{\omega_- z}+1 & l_b < z \end{cases} \tag{S20}$$

Furthermore, we consider a limiting situation with vanishing sized plasmid $l_b \to 0$ for S9 and S20 Eqs. The limit $l_b \to 0$ leads to $\chi = kN/(2l_b) \to \infty$ and $\varepsilon = \mathcal{D}N/(2l_b) \to \infty$. Even under such limit, steady surfing-on-wave solutions exist under a certain range of $k, \mathcal{D}$, and N. The limit of $U^{st}(z)$ and $U_z^{st}(z)$ for $|z| < l_b(\to 0)$ are obtained as

$$\lim_{l_b\to 0} U^{st}(z;v,k,l_b) = U^{st}(0;v,k,0) = 1 - \frac{kN}{\omega_+-\omega_-} = 1 - \frac{kN}{\sqrt{4+v^2}} \tag{S21}$$

$$\lim_{l_b\to 0} U_z^{st}(z;v,k,l_b) = U_z^{st}(0;v,k,0) = -\frac{kN}{2}\frac{\omega_++\omega_-}{\omega_+-\omega_-} = \frac{kN}{2}\frac{v}{\sqrt{4+v^2}}, \tag{S22}$$

where we used two limits: $\lim_{l_b\to 0}\chi\sinh(\omega_\pm l_b) = kN\omega_\pm/2$ and $\lim_{l_b\to 0}\chi[\cosh(\omega_\pm l_b)-1] = 0$. On the other hand, taking the limit of S9 Eq, we get

$$v = \lim_{l_b\to 0}\mathcal{D}N\int_{-L/2}^{L/2} dz \frac{U_z^{st}(z;v,k,l_b)}{U^{st}(z;v,k,l_b)}\frac{\theta(l_b-|z|)}{2l_b} = \mathcal{D}N\int_{-L/2}^{L/2} dz \frac{U_z^{st}(z;v,k,0)}{U^{st}(z;v,k,0)}\delta(z) = \mathcal{D}N\frac{U_z^{st}(0;v,k,0)}{U^{st}(0;v,k,0)}. \tag{S23}$$

Finally, we obtain a self-consistent equation in the vanishing size limit,

$$v = \mathcal{D}N\frac{U_z^{st}(0;v,k,0)}{U^{st}(0;v,k,0)} = \frac{\mathcal{D}kN^2}{2}\frac{v}{\sqrt{4+v^2}-kN}. \tag{S24}$$

S24 Eq has a trivial localized solution with $v = 0$ for any k and two solutions with $v \neq 0$ for $k > k_c$,

$$v = \pm\sqrt{\left[kN\left(\frac{\mathcal{D}N}{2}+1\right)+2\right]\left[kN\left(\frac{\mathcal{D}N}{2}+1\right)-2\right]} \tag{S25}$$

Here, $k = k_c := \frac{2}{N\left(\frac{\mathcal{D}N}{2}+1\right)}$ is a bifurcation point, below which two real solutions for the directed movement exist. Corresponding to Fig 2A and 2B in the main text, we show the phase diagram (S6A Fig), and the relationship between v and k (S6B Fig) in the case of $\mathcal{D} = 0.05, N = 40$.

REFERENCES

1. Nagayama M, Nakata S, Doi Y, Hayashima Y. A theoretical and experimental study on the unidirectional motion of a camphor disk. Physica D 2004;194:151–16.

2. Purwins HG, Bödeker HU, Liehr AW. Dissipative solitons in reaction-diffusion systems. In Dissipative solitons, Springer, 267–308.
