# Supplementary figures and images for: Chemophoresis engine: A general mechanism of ATPase-driven cargo transport
Source: PLoS Comput Biol. 2022 Jul 25;18(7):e1010324. doi: 10.1371/journal.pcbi.1010324 (PMC9363008; doi:10.1371/journal.pcbi.1010324)

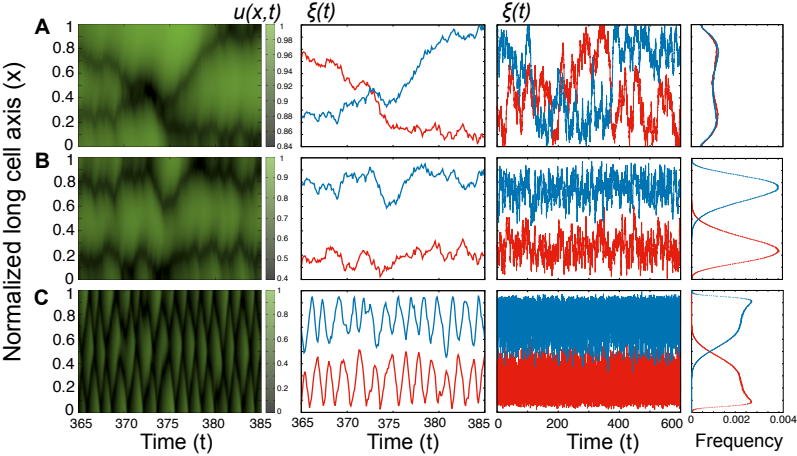

Supplement: S1 Fig — The dynamics change among stochastic switching, steady equi-positioning, and directed movement followed by oscillatory mode as χ increases among χ = 0.5 (A), χ = 2.5 (B), and χ = 10 (C) (two inner figures). The corresponding ParA-ATP pattern dynamics also change among stochastic switching, steady equi-positioning, and oscillatory waves (left). The oscillatory behavior of plasmids does not disrupt time-averaged equi-positioning. Steady multi-modal distributions of plasmids are sustained (Compare (B) right and (C) right). Kd = 0.1, ε = 5, and L = 5. The distributions (right) were generated using 107 samples over 105 time step. (PDF) [file pcbi.1010324.s003.pdf]

Normalized long cell axis (x)

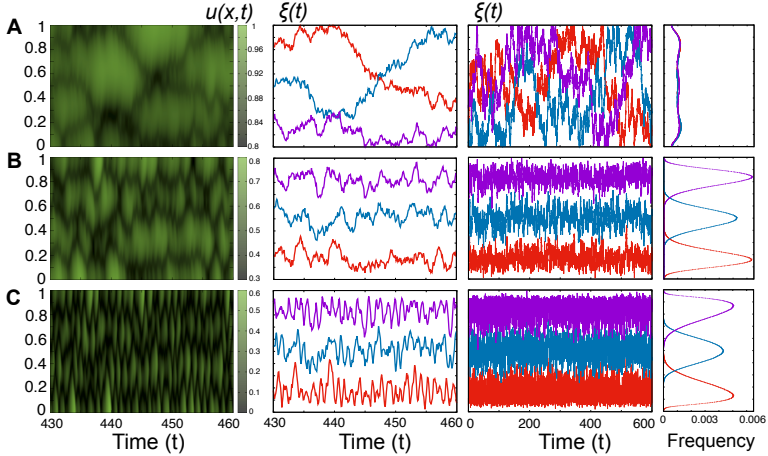

Supplement: S2 Fig — The dynamics change among stochastic switching, steady equi-positioning, and directed movement followed by oscillatory mode as χ increases among χ = 0.5 (A), χ = 2.5 (B), and χ = 10(C) (two inner figures). The corresponding ParA-ATP pattern dynamics also change among stochastic switching, steady equi-positioning, and oscillatory waves (left). The oscillatory behavior of plasmids does not disrupt time-averaged equi-positioning. Steady multi-modal distributions of plasmids are sustained (Compare (B) right and (C) right). Kd = 0.1, ε = 5, and L = 5. The distributions (right) were generated using 107 samples over 105 time step. (PDF) [file pcbi.1010324.s004.pdf]

**A** $u(x,t)$ 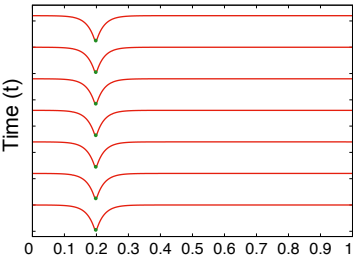Normalized long cell axis ( $x$ )**B** $u(x,t)$ 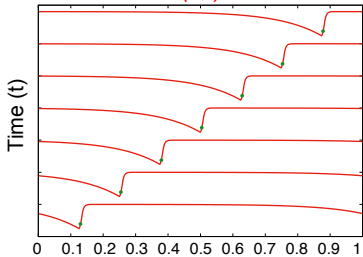Normalized long cell axis ( $x$ )

Supplement: S3 Fig — χ = 2.5 (A) and χ = 10 (B) with Kd = 0.001. The red lines show the spatial pattern of u(x, t) (=ParA-ATP) for each t. Green dots show a plasmid location for each t. In the former case (A), the plasmid maintains its location, whereas it surfs on the traveling wave of u(x, t) and moves unidirectionally in the latter (B). These results were compared with the analytical results in the main text and in Fig 2. ε = 5 and L = 40. (PDF) [file pcbi.1010324.s005.pdf]

$$\varepsilon\Delta\mu(v, \chi) - v$$

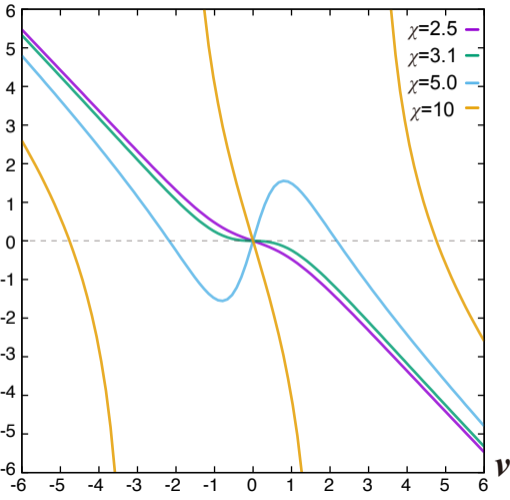

Supplement: S4 Fig — S9 Eq in S2 Text shows a pitch-fork bifurcation at χ = χc ∼ 3.1, and has three solutions for χ > χc. ε = 5 and L = 40. (PDF) [file pcbi.1010324.s006.pdf]

$\nu$ 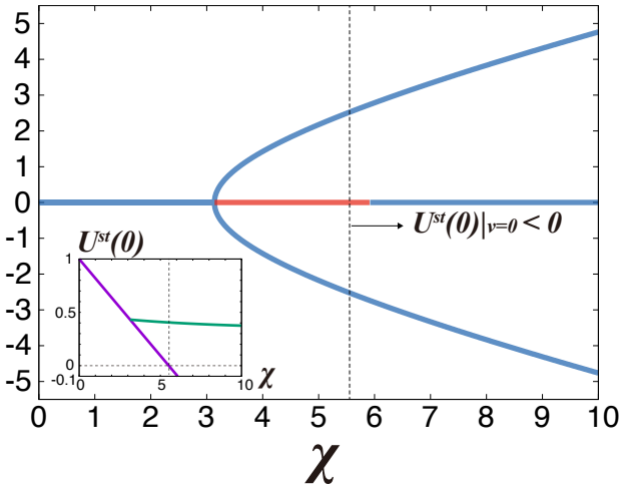

Supplement: S5 Fig — From tiny perturbations around the stationary solution U(z, t) = Ust(z) + eλtδU(z), and zξ(t) = eλtδzξ, the eigenvalues λ were computed as a function of χ. The stability of the plasmid-surfing pattern (blue line) and the instability of the plasmid-localized solution (red line) was confirmed for χ > χc in the absence of any positive real parameters of λ, Re[λ(v)] > 0, v ≠ 0. It seems to regain stability at χ larger than χ ∼ 5.9. However, such localized solution cannot be numerically realized for χ > 5.5 because Ust(0) becomes negative and the solution is unphysical at χ ∼ 5.5 (inset) as a result of breaking the approximation u(x) ≫ Kd. Therefore, the stable localized solution does not exist for χ≳5.5. (PDF) [file pcbi.1010324.s007.pdf]

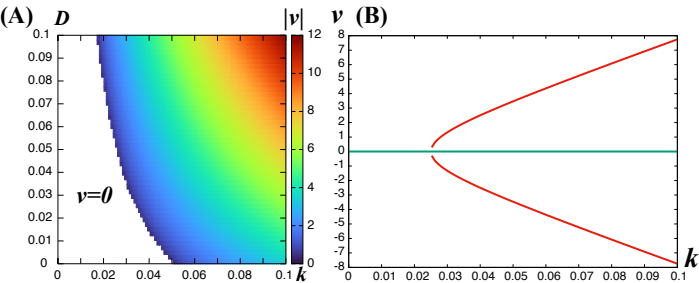

Supplement: S6 Fig — (A) Steady velocity (v) profile of plasmid movement for 0 < k < 0.1 and 0<D<0.1 for N = 40. (B) Relationship between v and k in analytical solutions for S25 Eq in S2 Text. Solutions for directed movement (|v| > 0) emerge at k = kc = 0.025 as a result of a supercritical pitchfork bifurcation, whereas a solution for localization (v = 0) exists over 0 ≤ k ≤ 0.1 in D=0.05, and N = 40. (PDF) [file pcbi.1010324.s008.pdf]

**(A)****(A1)**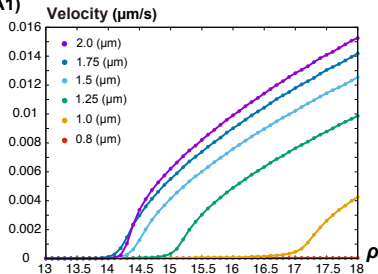**(B)****(B1)**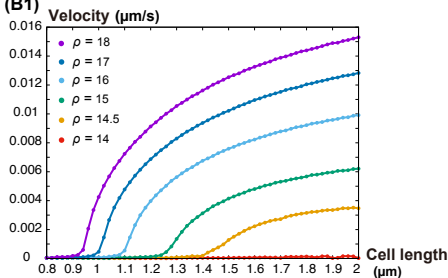**(A2)**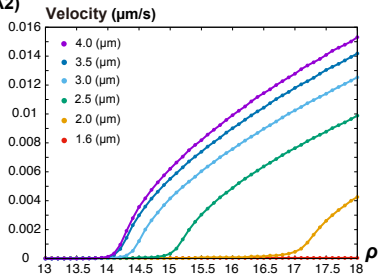**(B2)**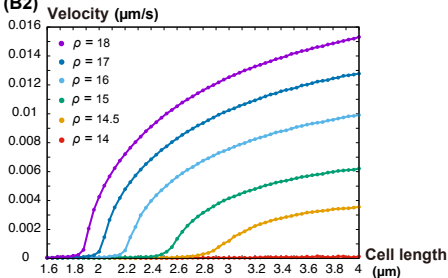

Supplement: S7 Fig — (A) Relationship between time-averaged velocity |v| and ρ at cell lengths with 0.8, 1.0, 1.25, 1.5, 1.75, and 2.0 for a single-plasmid case M = 1 (A1), and 1.6, 2.0, 2.5, 3.0, 3.5, and 4.0 for a two-plasmid case M = 2 (A2). (B) Relationship between time-averaged velocity |v| and the cell length at ρ = 14, 14.5, 15, 16, 17, and 18 for both M = 1 (B1) and M = 2 (B2). See S1 Table for the other model parameters. (PDF) [file pcbi.1010324.s009.pdf]

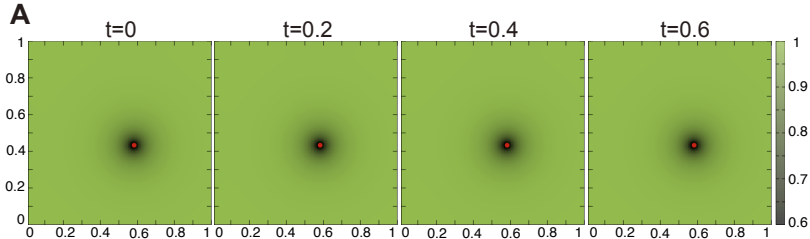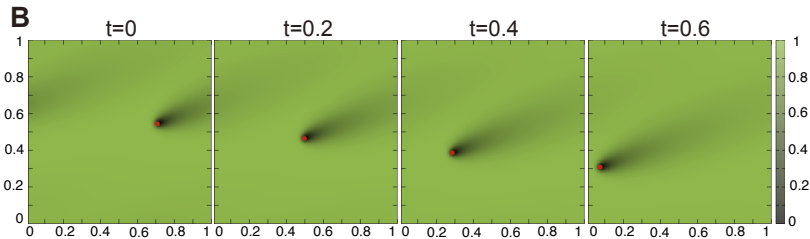

Supplement: S8 Fig — Successive snapshots are shown for χ = 10 (A) and χ = 50 (B). In the former case (A), the plasmid (red circle) maintains its location at a minimum symmetrical shape of u(r, t) (=ParA-ATP, green scale), whereas it moves unidirectionally with an asymmetrical pattern of u(r, t) in the latter (B). Kd = 0.001, lb = 0.2, ε = 5, and L2 = 10 × 10. (PDF) [file pcbi.1010324.s010.pdf]

**A**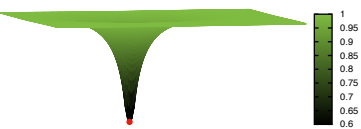**B**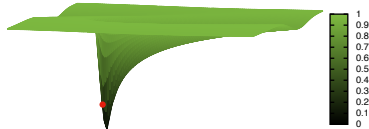

Supplement: S9 Fig — Snapshots are shown for χ = 10 (A) and χ = 50 (B). In the former case (A), u(r, t) (=ParA-ATP, green scale) is symmetrical, and the plasmid (red circle) is located at its minimum. In contrast, for the latter (B), the symmetry of u(r, t) is broken, indicative of a traveling wave. The minimum of the asymmetrical u(r, t) is positioned at a location shifted from where the plasmid lies, suggesting that the plasmid in “surfing” on the traveling wave.” Kd = 0.001, lb = 0.2, ε = 5, and L2 = 10 × 10. (PDF) [file pcbi.1010324.s011.pdf]
