# Supplementary material for: Chemophoresis engine: A general mechanism of ATPase-driven cargo transport
Source: PLoS Comput Biol. 2022 Jul 25;18(7):e1010324. doi: 10.1371/journal.pcbi.1010324 (PMC9363008; doi:10.1371/journal.pcbi.1010324)
Supplement: S1 Table — (PDF) [file pcbi.1010324.s012.pdf]

S1 TABLE

Parameter	Explanation	Reported value	Chosen value	Normalized parameter	Reference
L	Cell length	0.8 - 4.0 µm	0.8 - 4.0 µm	$\tilde{L} = L/l$	(1)
l_b	Radius of plasmid (partition complex, PC)	~ 0.075 µm	0.08 µm	$\tilde{l}_b = l_b/l$	(1, 2)
D_u	ParA diffusion coefficient	0.001 - 0.01 µm^2/s	0.004 µm^2/s		(3)
D_ξ	Plasmid diffusion coefficient	~0.001 µm^2/s	0.001 µm^2/s		(4, 5)
N_{ParA}	Number of ParA dimers per cell	100 - 4000			(4–6)
N	Number of binding sites (ParB)	500 - 1000 /PC			(6, 7)
u_0	ParA dimer density (reference density)	(determined from N_{ParA})	1.0 µM	$\tilde{u}_0 = 1$	This study
ρ	ParB density in PC		13 - 18 $\times u_0$ µM	$\tilde{\rho} = \rho/u_0$	This study
a	Spontaneous dissociation rate of ParA-ATP from nucleoid	1.5 - 6.0 min^{-1}	1.5 min^{-1}	$\tilde{a} = 1$	(8, 9)
k	Hydrolysis rate constant	~ 1.0 min^{-1}	1.5 min^{-1}	$\tilde{k} = k/a$	(9)
$K_d = k_-/k_+$	Dissociation constant for ParA and ParB	~ 0.24 µM	0.25 µM	$\tilde{K}_d = K_d/u_0$	(9)
τ	Reference time scale $\tau = 1/a$		40 s	$\tilde{\tau} = 1$	This study
l	Reference length scale $l = \sqrt{D_u \tau} = \sqrt{D_u/a}$		0.4 µm	$\tilde{l} = 1$	This study

REFERENCES

1. Le Gall A, Cattoni DI, Guilhas B, Mathieu-Demazière C, Oudjedi L, Fiche J-B, et al. Bacterial partition complexes segregate within the volume of the nucleoid. Nat Commun 2016;7:12107.
2. Sanchez A, Cattoni DI, Walter J-C, Rech J, Parmeggiani A, Nollmann M, Bouet J-Y. Stochastic self-assembly of ParB proteins builds the bacterial DNA segregation apparatus. Cell Syst 2015;1:163.
3. Surovtsev IV, Lim HC, Jacobs-Wagner C. The slow mobility of the ParA partitioning protein underlies its steady-state patterning in Caulobacter. Biophys J 2016;110:2790–2799.
4. Ietswaart R, Szardenings F, Gerdes K, Howard M. Competing ParA structures space bacterial plasmids equally over the nucleoid. PLoS Comput Biol 2014;10:e1004009.
5. Surovtsev IV, Campos M, Jacobs-Wagner C. DNA-relay mechanism is sufficient to explain ParA-dependent intracellular transport and patterning of single and multiple cargos. Proc Natl Acad Sci USA 2016;113:E7268–E7276.
6. Lim HC, Surovtsev IV, Beltran BG, Huang F, Bewersdorf J, Jacobs-Wagner C. Evidence for a DNA-relay mechanism in ParABS-mediated chromosome segregation. Elife 2014;3:e02758.
7. Bouet J-Y, Rech J, Egloff S, Biek DP, Lane D. Probing plasmid partition with centromere-based incompatibility. Mol Microbiol 2005;55:511.
8. Vecchiarelli AG, Hwang LC, Mizuuchi K. Cell-free study of F plasmid partition provides evidence for cargo transport by a diffusion-ratchet mechanism. Proc Natl Acad Sci USA 2013;110:E1390–E1397.
9. Taylor JA, Seol Y, Budhathoki J, Neuman KC, Mizuuchi K. CTP and parS coordinate ParB partition complex dynamics and ParA-ATPase activation for ParABS-mediated DNA partitioning. eLife 2021;10:e65651.
