# Supplementary material for: Chemophoresis engine: A general mechanism of ATPase-driven cargo transport
Source: PLoS Comput Biol. 2022 Jul 25;18(7):e1010324. doi: 10.1371/journal.pcbi.1010324 (PMC9363008; doi:10.1371/journal.pcbi.1010324)
Supplement: Attachment — Submitted filename: Kaneko_PLOS.pdf [file pcbi.1010324.s013.pdf]

In this manuscript, Sugawara and Kaneko have proposed a model based on chemophoresis and carried out theoretical analysis of the model to reveal the mechanism underlying the emergence of traveling wave and pole-to-pole oscillation of ATPase concentration for ParABS system. The authors simplified the system, such that a cargo (a plasmid on nucleoid) has N binding sites where ParA-ATP can bind and hydrolyze. Due to externally sustained concentration gradients, there would be more frequent binding and catalysis at space with higher Par-ATP concentration (chemical potential), and the resulting chemophoretic force drives the cargo towards the region with higher ParA-ATP concentration. It was further shown that at sufficeintly high reactivity, parameterized by $\chi(=kN/V)$, the traveling wave with $|v| \neq 0$ or pole-to-pole oscillation can arise.

The authors propose that their chemophoresis engine model works as a universal principle of hydrolysis-driven intracellular cargo transport.

The mathematical structure of the equations representing the physics underlying ParABS system and the authors corresponding analysis based on the theories of dynamical system are elegant. I personally enjoyed reading the paper.

I, however, feel that the presentation of the paper is a bit condensed, and also that some clarifications are required.

1. Though the authors are discussing the dynamical behavior of the system over the entire parameter space, physical relevance of some parameters should be justified against the real physical situations.

— Dynamical phase diagram of $|v|$(emergence of traveling wave) as a function of ε and χ suggests that the traveling wave emerges only when special conditions are met. For instance $|v| \neq 0$ is obtained only when $\varepsilon\left(\equiv \frac{D_\xi}{D_u}\frac{N}{2l_b}\right) \gtrsim 1$ and χ is sufficiently large. However, I would argue that $D_\xi/D_u \ll 1$ under the typical situation due to the apparent size difference between the plasmid and ParA-ATP. To compensate this, $N/2l_b$ should be sufficiently large, in order for us to observe $|v| \neq 0$. Please comment on the typical range of ε in ParABS system.

— The catalytic rate k is ParB concentration dependent. What is the biophysically meaningful range of k (or χ)?

— The authors set K_d=0.1, 0.001 and m=1, but I believe there are already biochemical measurements available on K_d and m.

2. The authors' idea of chemophoretic force driven cargo transport is interesting; yet, saying this particular mechanism as the **universal principle of cargo transport** sounds like too much of exaggeration. As far as Vecchiarelli et al's experiment is concerned, the authors' work offer nice explaination. But, when it comes to real biological systems, there are too many unknowns inside even in bacteria. First, are there real evidences that concentration gradients of ParA-ATP are constantly maintained? Second, DNA polymerase, RNA polymerase, ribosome are essentially the molecular motors that transduce the chemical free energy acquired from chemical transformation of substrates to unidirectional motion. Their motion can potentially be coupled with ParABS system. Given that there are many kinds of molecular components that give rise to unidirectional motion, it is not clear how the authors can assert that theirs is a universal mechanism in cargo transport, ruling out other mechanisms.

Furthermore, for eukaryotes, it is already well known and have been studied over many decades by many researchers that cargo transports inside cells and axons are mediated by the actions of ATP-

consuming molecular motors (kinesin, dynein, myosin, etc) that translocate along cytoskeletal filaments that spread throughout the cell. This basic fact is not even mentioned in the manuscript.
