# Supplementary material for: Chemophoresis engine: A general mechanism of ATPase-driven cargo transport
Source: PLoS Comput Biol. 2022 Jul 25;18(7):e1010324. doi: 10.1371/journal.pcbi.1010324 (PMC9363008; doi:10.1371/journal.pcbi.1010324)
Supplement: Attachment — Submitted filename: ResponsesToReviewers_PLCB_20220504_submit.pdf [file pcbi.1010324.s014.pdf]

Point-by-point responses to comments made on the previous version of the manuscript (The original comments are italicised and shaded.)

All comments of Reviewer #1:

In this manuscript, Sugawara and Kaneko have proposed a model based on chemophoresis and carried out theoretical analysis of the model to reveal the mechanism underlying the emergence of traveling wave and pole-to-pole oscillation of ATPase concentration for ParABS system. The authors simplified the system, such that a cargo (a plasmid on nucleoid) has N binding sites where ParA-ATP can bind and hydrolyze. Due to externally sustained concentration gradients, there would be more frequent binding and catalysis at space with higher Par-ATP concentration (chemical potential), and the resulting chemophoretic force drives the cargo towards the region with higher ParA-ATP concentration. It was further shown that at sufficeintly high reactivity, parameterized by $\chi(=kN/V)$, the traveling wave with $|v| \neq 0$ or pole-to-pole oscillation can arise. The authors propose that their chemophoresis engine model works as a universal principle of hydrolysis-driven intracellular cargo transport.

The mathematical structure of the equations representing the physics underlying ParABS system and the authors corresponding analysis based on the theories of dynamical system are elegant. I personally enjoyed reading the paper.

I, however, feel that the presentation of the paper is a bit condensed, and also that some clarifications are required.

1. *Though the authors are discussing the dynamical behavior of the system over the entire parameter space, physical relevance of some parameters should be justified against the real physical situations.*

   — Dynamical phase diagram of $|v|$ (emergence of traveling wave) as a function of ε and χ suggests that the traveling wave emerges only when special conditions are met. For instance $|v| \neq 0$ is obtained only when $\varepsilon \left(\equiv \frac{D_\xi}{D_u}\frac{N}{2l_b}\right) \gtrsim 1$ and χ is sufficiently large. However, I would argue that $D_\xi/D_u \ll 1$ under the typical situation due to the apparent size difference between the plasmid and ParA-ATP. To compensate this, $N/2l_b$ should be sufficiently large, in order for us to observe $|v| \neq 0$. Please comment on the typical range of ε in ParABS system.

— The catalytic rate k is ParB concentration dependent. What is the biophysically meaningful range of k (or χ)?

— The authors set $K_d = 0.1, 0.001$ and $m = 1$, but I believe there are already biochemical measurements available on K_d and m.

2. *The authors' idea of chemophoretic force driven cargo transport is interesting; yet, saying this particular mechanism as the* ***universal principle of cargo transport*** *sounds like too much of exaggeration. As far as Vecchiarelli et al's experiment is concerned, the authors' work offer nice explaination. But, when it comes to real biological systems, there are too many unknowns inside even in bacteria. First, are there real evidences that concentration gradients of ParA-ATP are constantly maintained? Second, DNA polymerase, RNA polymerase, ribosome are essentially the molecular motors that transduce the chemical free energy acquired from chemical transformation of substrates to unidirectional motion. Their motion can potentially be coupled with ParABS system. Given that there are many kinds of molecular components that give rise to unidirectional motion, it is not clear how the authors can assert that theirs is a universal mechanism in cargo transport, ruling out other mechanisms.*

   Furthermore, for eukaryotes, it is already well known and have been studied over many decades by many researchers that cargo transports inside cells and axons are mediated by the actions of ATP-consuming molecular motors (kinesin, dynein, myosin, etc) that translocate along cytoskeletal filaments that spread throughout the cell. This basic fact is not even mentioned in the manuscript.

Point #1-1

I, however, feel that the presentation of the paper is a bit condensed, and also that some clarifications are required.

1. *Though the authors are discussing the dynamical behavior of the system over the entire parameter space, physical relevance of some parameters should be justified against the real physical situations.*

   — Dynamical phase diagram of $|v|$ (emergence of traveling wave) as a function of ε and χ suggests that the traveling wave emerges only when special conditions are

met. For instance $|v| \neq 0$ *is obtained only when* $\varepsilon \left(\equiv \frac{D_\xi}{D_u}\frac{N}{2l_b}\right) \gtrsim 1$ *and* χ *is sufficiently large. However, I would argue that* $D_\xi/D_u \ll 1$ *under the typical situation due to the apparent size difference between the plasmid and ParA-ATP. To compensate this,* $N/2l_b$ *should be sufficiently large, in order for us to observe* $|v| \neq 0$*. Please comment on the typical range of* ε *in ParABS system.*

Thank you for your useful comments. We have estimated parameter values capturing *in vivo* experiments, as summarized in S1_Table.

(1) About D_ξ/D_u

In ideal diffusion in aqueous solution, where the diffusion coefficient is proportional to the particle size, $D_\xi/D_u \ll 1$ would be expected as you commented. However, the present situation is different; D_ξ and D_u are "effective" diffusion coefficients, since the plasmid and ParA-ATP bind on the nucleoid and move on a 2-dimensional crowded surface. In fact, previous experimental studies have reported $D_\xi \sim 0.001(\mu m^2/s)$ ([33,35], S1_Table) and $D_u = 0.001 \sim 0.01(\mu m^2/s)$ ([34], S1_Table), suggesting that D_ξ/D_u is in fact of the order of 0.1~ 1; the experimental data do not support $D_\xi/D_u \ll 1$. Therefore, we have shown the results in (newly added) S7_Fig, using the value $D_\xi/D_u = 0.25$ as a value capturing the intracellular condition, quantitatively reproducing the directed movement therein. On the other hand, in Figs. 1 and 2 of the main text we reproduced the directed motion for the smaller value, $D_\xi/D_u = 0.05$, demonstrating that our theory can work even in the case of $D_\xi/D_u \ll 1$.

(2) About ε.

$\varepsilon = (D_\xi/D_u)(N/2\tilde{V})$ depends on the (normalized) local concentration of ParB dimer that forms a complex (partition complex, PC) with the plasmid, $\tilde{\rho} = N/2\tilde{V} = \rho/u_0$; Here, N is the number of ParB dimer molecules in PC, $\rho = N/2V$ is the local concentration, and u_0 is a (reference) intracellular ParA-ATP concentration. From the number of intracellular ParA molecules $N_{ParA} \sim 1000$ [33, 35] and the cell volume of bacteria $\sim 1.0(\mu m^3)$, u_0 can be evaluated as $u_0 \sim 1.0(\mu M)$. N is very large in general, suggested to be $N \sim 200$ in early experiments [72, 74]. Although there are no reports of intracellular

ρ values, ρ is expected to be large as well because the ParB dimers are localized in the PC. We have added new numerical results for intracellular conditions presented in S7_Fig by using $\rho = 13{\sim}18\ (\mu M)$. Considering the above information, values of $\varepsilon = (D_\xi/D_u)\tilde{\rho} = 3.25{\sim}4.5$ were used in S7_Fig. Also, the parameter values estimated from experiments are summarized in newly added S1_Table.

Point #1-2

— The catalytic rate k is ParB concentration dependent. What is the biophysically meaningful range of k (or χ)?

Thank you for your useful suggestions.

(1) About k.

The hydrolysis rate constant k has been reported by recent *in vitro* experiments to be $k \sim 1.0\ (min^{-1})$ according to [75]; $k = 1.5\ (min^{-1})$ was used to recapitulate intracellular conditions (Please see newly added S7_Fig and S1_Table).

(2) About χ.

As you point out, the maximum rate of (normalized) hydrolysis $\chi = \tilde{k}(N/2\tilde{V})$ depends on the (normalized) local concentration of ParB dimer in PC, $\tilde{\rho} = N/2\tilde{V} = \rho/u_0$, and the normalized hydrolysis rate constant $\tilde{k} = k/a$; Here, a is a spontaneous dissociation rate constant of ParA-ATP, reported to be 1.5 ~ 6.0 (min^{-1}) [21, 75]. Based on the discussion in #1-1, $u_0{\sim}1.0(\mu M)$ and $\rho = 13{\sim}18(\mu M)$ were used in the numerical results for intracellular conditions presented in S7_Fig and S1_Table added in the revised version; Considering the above information, values of $\chi = \tilde{k}\tilde{\rho} = 13{\sim}18$ were used in S7_Fig ($\tilde{k} = 1.0\ and\ u_0 = 1.0(\mu M)$).

Point #1-3

— The authors set $K_d = 0.1, 0.001$ and $m = 1$, but I believe there are already biochemical measurements available on K_d and m.

(1) About Hill coefficient $\boldsymbol{m}$.

Since ParA-ATP always exists as a dimer on the nucleoid, it is reasonable to simply consider $m = 1$ for a hydrolysis reaction in a spatially limited space around a plasmid. We added a remark on this in the part of numerical methods in **Models** as follows: **"Since ParA-ATP always exists as dimer on a nucleoid, it is reasonable to simply consider a hydrolysis reaction without any cooperativity in a spatially limited space around a plasmid. Therefore, the Hill coefficient $\boldsymbol{m}$ was fixed as $\boldsymbol{m = 1}$ in all the simulations."**

(2) About the dissociation constant $\boldsymbol{K_d}$.

In general, K_d can be measured by conventional biochemical methods, but the situation is more complicated because K_d in the ParABS system reflects the interaction of ParA-ATP and ParB (as well as *parS*) on the 2D nucleoid surface. However, we were able to extract information from a recent report: in an *in vitro* experiment by Taylor et al. [75], the dependence of the hydrolysis rate on ParB (but not ParA-ATP) concentration was measured for the reaction $(ParA - ATP)_2 + hParB \overset{k_\pm}{\leftrightarrow} Y \overset{k}{\rightarrow} 2ParA + hParB + 2ADP$ and the Michaelis-Menten constant K_M was evaluated to be $K_M \sim 0.24(\mu M)$ at ParA concentration of $1.0(\mu M)$; In addition, $h \sim 1.2$ and $k \sim 1.0\ (min^{-1})$ (Fig. 2 F and Table1 in [75]). On the other hand, when considering the dependence on ParA-ATP concentration ($= u(\boldsymbol{r})$) in our model, we can adopt the same value of K_d since $K_d = k_+/k_-$ is expected to remain unchanged in the case; Note that we assume $K_M \sim K_d$ through our study.

Therefore, the results obtained using the value of $K_d = 0.25(\mu M)$ as the intracellular condition are shown in S7_Fig. It should be noted that the normalized value $\widetilde{K}_d = K_d/u_0$ used in the numerical calculations is $\widetilde{K}_d = 0.25$ from $u_0 = 1.0(\mu M)$.

In the revised manuscript, to reflect referee comments #1-1 ~ #1-3, we have therefore added the simulation results with parameters capturing intracellular conditions and explanations (S7_Fig, S1_Table,) and revised the concerning part of the manuscript as follows:

[Page 8~9]

Chemophoresis engine can work in *in vivo* conditions

From the normalized equations for chemphoresis engine (Eqs 3 and 4), we here confirmed that plasmid surfing on the traveling wave emerged through a symmetry-breaking transition with the change of χ and ε (Fig 2). However, it remains unclear whether the above results can be quantitatively reproduced for the parameter values reported experimentally in bacterial cells. To quantitatively confirm that the directed motion is self-organized by the chemophoresis engine, we ran the simulations again with parameters capturing *in vivo* conditions (S1_Table), as described below.

A recent study suggests that ParB localizes around plasmids by a ParA-ATP dependent phase separation mechanism to form PCs as droplets, accompanied by an enhanced hydrolysis activity of ParA-ATP through the increase in local density of ParB within the PCs, finally leading to a successful plasmid partition [76, 77]. As a simple demonstration, we carried out the simulation by simultaneously changing the maximum rate of ParA-ATP hydrolysis $\chi = kN/2l_b$ and the strength of the chemophoresis force $\varepsilon = \mathcal{D}N/2l_b$ under the control of the local density of ParB on a PC, $\rho = N/2l_b$. We increased the cell length, which may reflect the cell cycle progression, whereas we adopted the parameters capturing *in vivo* conditions listed in S1_Table. We changed ρ and the cell length $= L \times l$ with L as a normalized system size, scaled by $l = \sqrt{D_u \tau} = \sqrt{D_u/a} = 0.4(\mu m)$, whereas ρ is a dimensionless relative density normalized by the cytoplasmic one of ParA-ATP assigned to u_0 (See also Models for normalized parameters and S1_Table for *in vivo* parameter values).

We have performed simulations for the single plasmid ($M = 1$, S7_Fig upper) and the two-plasmid ($M = 2$, S7_Fig bottom) cases using Neumann boundary condition as in Fig 1. We investigated how the directed (or pole-to-pole oscillatory) movement of plasmids emerges with the increase in ρ, by calculating time-averaged values of velocity for

different cell lengths (S7_Fig A). The directed motion appeared at a $\rho = \rho_c$ for cell lengths greater than $0.8(\mu m)$ for $M = 1$ (S7_Fig A1) and $1.6(\mu m)$ for $M = 2$ (S7_Fig A2), demonstrating that the chemophoresis engine can recapitulate plasmid surfing even in *in vivo* conditions. Interestingly, the bifurcation points differ by different cell lengths; that is, the emergence of directed movement can be cell-length dependent. This bifurcation to the directed motion against the cell length occurs for both $M = 1$ and $M = 2$ (S7_Fig B1 and B2). In addition, the bifurcation diagram for $M = 1$ coincides with that for $M = 2$ when plotted as a function of (cell-length)$/M$ both for S7_Fig A and S7_Fig B, suggesting that controlling the inter-plasmid distance is important for the emergence of directed motion.

Point #1-4

First, are there real evidences that concentration gradients of ParA-ATP are constantly maintained?

Thank you for your helpful suggestion.

Directed movement due to a steady-state concentration gradient (traveling wave) was proposed in the *in vitro* experiments of Vecchiarelli et al [27]. On the other hand, there are several reports suggesting the existence of a concentration gradient in the *in vivo* experiments of plasmid partition [24, 25, 34, 73], also even though it may be hard to confirm that the gradient is constantly maintained.

We have added a remark in the introduction section of the main text as follows: "**Indeed, there are recent reports suggesting the existence of a concentration gradient in the *in vivo* experiments [26, 27]**".

Point #1-5

Given that there are many kinds of molecular components that give rise to unidirectional motion, it is not clear how the authors can assert that theirs is a universal mechanism in cargo transport, ruling out other mechanisms.

Thank you for your comment.

As you point out, there are many known molecular mechanisms that lead to directed movement, and hence the statement of chemophoresis as if it were the only universal principle is certainly an exaggeration. On the other hand, one third of prokaryotes encode the ParABS system, and archaea also have it, suggesting that the ParABS system is a common molecular mechanism for chromosome segregation and organelle arrangement of ancient origin; Therefore, we propose that the chemophoresis engine, which describes ParABS, works as a general mechanism, although it has not been reported in eukaryotes so far.

With respect to the universality of the ParABS system, the third paragraph of the introduction section contains the above example:

"ParA ATPase is an evolutionarily conserved protein which has many homologs [43-46]. Representative examples of its family are McdA/McdB ATPases controlling the equidistribution of carboxysomes along a long cell axis in cyanobacteria [47-49], ParC/PpfA ATPases that regulate intracellular positions of chemotaxis protein clusters [50-52], ..."

In addition, we have modified statements for "universal principle" as follows, emphasizing that it is "general mechanism" so that the reader does not misunderstand.

(1) **[Title]** Chemophoresis Engine: a General Mechanism of ATPase-driven Cargo Transport

(2) **[Abstract]** Finally, we discuss the chemophoresis engine as one of the general mechanisms of hydrolysis-driven intracellular transport.

(3) **[Author summary]** We propose that this chemophoresis engine works as a general mechanism of hydrolysis-driven intracellular transport.

(4) **[Discussion]** We propose the chemophoresis engine as a general mechanism for hydrolysis-driven cargo transports in cells.

Point #1-6

cargo transports inside cells and axons are mediated by the actions of ATP-consuming molecular motors (kinesin, dynein, myosin, etc) that translocate along cytoskeletal filaments that spread throughout the cell. This basic fact is not even mentioned in the manuscript.

Thank you for your helpful comment.

As you point out, the common mechanism of intracellular transport is the cargo transport mechanism by cytoskeleton and molecular motors, which should be emphasized to avoid any misunderstanding. We have therefore modified the red part of the first paragraph in the introduction section as follows:

"**Cell polarity regulates the direction of intracellular transport for cargos, such as organelles and macromolecules, by taking advantage of chemical gradients sustained with the aid of ATP or GTP hydrolysis.**

For example, it is well known that eukaryotic cell polarity factors, such as Rho, GTPase, and Cdc42, regulate the orientation of cytoskeleton members so that molecular motors can carry cargo directionally on the cytoskeleton, contributing to cell movement [4], cell growth [5], and axon guidance [6].

Although the transport by the cytoskeleton is one of the most commonly observed mechanisms, the transport directly mediated by chemical gradient, if its existence is confirmed, should be of importance as a general mechanism for cargo transport as well, which we refer to as cargo chemotaxis here."

Finally, we have added recent references in the revised manuscript as follows:

[27] Le Gall A, Cattoni DI, Guilhas B, Mathieu-Demazière, Oudjedi L, Fiche J-B, Rech J, Abrahamsson S, Murray H, Bouet J-Y, Nollmann M. Bacterial partition complexes segregate within the volume of the nucleoid. Nat Commun 2016;7:12107.

[38] Hu L, Rech J, Bouet J-Y, Liu J. Spatial control over near-critical-point operation ensures fidelity of ParABS-mediated DNA partition. Biophys J 2021;120:3911–3924.

[43] Burkart T, Wigbers MC, Würthner L, Frey E. Control of protein-based pattern formation via guiding cues. arXiv preprint arXiv:2202.10091.

[73] Sanchez A, Cattoni DI, Walter J-C, Rech J, Parmeggiani A, Nollmann M, Bouet J-Y. Stochastic self-assembly of ParB proteins builds the bacterial DNA segregation apparatus. Cell Syst 2015;1:163.
[74] Bouet J-Y, Rech J, Egloff S, Biek DP, Lane D. Probing plasmid partition with centromere-based incompatibility. Mol Microbiol 2005;55:511.
[75] Taylor JA, Seol Y, Budhathoki J, Neuman KC, Mizuuchi K. CTP and parS coordinate ParB partition complex dynamics and ParA-ATPase activation for ParABS-mediated DNA partitioning. eLife 2021;10:e65651.
[76] Guilhas B, Walter J-C, Rech J, David G, Walliser NO, Palmeri J, Mathieu-Demaziere C, Parmeggiani A, Bouet J-Y, Le Gall A, Nollmann M. ATP-driven separation of liquid phase condensates in bacteria. Mol Cell 2020;79:293–303.
[77] Biedzinski S, Parmar B, Weber SC. Beyond equilibrium phase diagrams: enzymatic activity shakes up bacterial condensates. Mol Cell 2020;79:205–206.

We thank you again for the clarification, which became clearer after we followed your suggestion in our discussion.

All comments of Reviewer #2:

Sugawara and Kaneko published the first theory paper on Chemophoresis a decade ago. Since then, the mathematical modeling studies from other labs have greatly extended this concept and applied to plasmid partition along with other cargo positioning in bacteria in several systems, both in vivo and in vitro. In this manuscript, Sugawara and Kaneko formulated the mathematical framework of chemophoresis, perhaps more vigorously. Again, they applied the model to ParA-mediated plasmid partition and recapitulated the different patterns such as plasmid oscillation and equi-distancing, which have been explained by other models as the authors cited. Overall, the theoretical work is solid and potentially transformative, in particular, in light of a general mechanism for cellular positioning. This work points out a potentially new mechanism of cargo positioning inside cell and thus holds a great interest in the field of cell motility and theory.

However, I fail to see what the new thing is in the current version as compared to their published work in Biophysics, nor how the work on plasmid partition can be extended to other systems as a general mechanism. I feel that the work as it currently stands may be more suitable for a specialized journal. On the other hand, the writing of this draft may mask something important. For instance, did the authors derive a new analytical formula/a more general mathematical model to predict different mobility patterns & delineate the conditions that cell can use this mechanism for transport and position cargos? If so, I'd suggest the authors to 1) make these points very explicit, and 2) discuss what can be explained by this work but not by others. And I would be more than happy to review it again.

Point #2-1

However, I fail to see what the new thing is in the current version as compared to their published work in Biophysics

Thank you for your comment.

We made the new findings of this study clear in our comment to Point #2-3 **(1)**, in terms of the differences from our present work. Please see the response to Point #2-3 **(1)**.

Point #2-2

However, I fail to see ..., nor how the work on plasmid partition can be extended to other systems as a general mechanism.

Thank you for your comment.

Our theory is derived from macroscopic thermodynamics under nonequilibrium conditions, and although we have applied it here to the ParABS system, it is general enough to be independent of individual microscopic models constructed for each molecular mechanism. In the present study, the mathematical model of the chemophoresis engine is constructed only by extracting the essential parts of the phenomena, so it can be applied directly to other systems with common reaction mechanisms such as hydrolysis. Indeed, it has been reported in *in vitro* experiments that the directed motion of a silica bead (with sizes of $0.5(\mu m)$ and $5.0(\mu m)$) is self-driven by the RNA gradient which RNA hydrolysis on the bead generates [85]; The authors later termed this phenomenon "autochemophoresis" [86].

In addition, we can give another example of possible applications: membraneless organelles (composed of RNA and proteins), which have been actively studied in recent years, are known to form droplets by a phase-separation mechanism and dissolve by ATP hydrolysis on the other hand [Hondele et al. Nature 2019]. Interestingly, its physicochemical mechanism is similar to that of the (phase-separation dependent) ParABS-driven plasmid partition [76, 77]. By focusing on the common mechanism of droplet dissolution by stimulated ATP hydrolysis, our theory could be applied to non-equilibrium phase separation in eukaryotic cells (especially in the cell nucleus, where no cytoskeleton is reported).

We added a remark on the above extension to other systems in **Discussion** as follows:

"**Our theory is derived from macroscopic thermodynamics under nonequilibrium conditions, and although we have applied it here to the ParABS system, it is general enough to be independent of individual microscopic models constructed for each molecular mechanism. In the present study, the mathematical model of the chemophoresis engine is constructed only by extracting the essential parts of the phenomena, so it can be applied directly to other systems with common reaction**

mechanisms such as hydrolysis. Indeed, it has been reported in *in vitro* experiments that the directed motion of a micro-sized bead is self-driven by the RNA gradient which RNA hydrolysis on the bead generates [85]; The authors later termed this phenomenon "autochemophoresis" [86].

Point #2-3

On the other hand, the writing of this draft may mask something important. For instance, did the authors derive a new analytical formula/a more general mathematical model to predict different mobility patterns & delineate the conditions that cell can use this mechanism for transport and position cargos? If so, I'd suggest the authors to 1) make these points very explicit,

Thank you for your comments.

(1) About the findings in the present study

The present model is an extension of a previous study (Sugawara and Kaneko BIOPHYSICS 2011, [3]) and aims to show that the chemophoresis engine works as a general, concentration-gradient driven mechanism, by reproducing and predicting (even quantitatively) a broader range of phenomena in plasmid partition. Our earlier model that neglected plasmid size for simplicity [3] was too unrealistic in reproducing the *in vitro* experiments by Vecchiarelli et al. PNAS 2014 [27] and reports of intracellular plasmid partition [24, 25, 34, 73]. By considering plasmids with a finite size ($= l_b$), we have now reproduced the directed movement and pole-to-pole oscillations of plasmids (and also the equi-positioning as in the previous result [3]) observed in *in vivo* and *in vitro* experiments, whereas we have made additional predictions. The new findings reported in the present manuscript specifically include:

(a) By using dynamical systems theory, we have shown mathematically and numerically that the symmetry of the ParA-ATP pattern is spontaneously broken and that the directed movement of a plasmid is self-organized through a pitchfork bifurcation (Figs 1 & 2, S1_Fig ~S5_Fig, S8_Fig & S9_Fig). We also showed analytically that the directed movement occurs even in the zero-size limit ($l_b \to 0$) of the present model (S6_Fig,

newly added results). These were not reported in the earlier paper [3]. In this revision, we have added new results as S6_Fig.

(b) We found that the directed motion of the plasmid depends on l_b (Fig. 2D): the velocity of the directed movement decreases monotonically with increasing l_b, and at a critical value of l_b, an inverse pitch-fork bifurcation occurs, eventually leading to a unique solution for $v = 0$. This result suggests that plasmids (or beads) with large l_b cannot generate a sharp concentration gradient and that the resulting chemophoretic forces are not sufficient to execute self-driven directed motion.

(c) Chemophoresis is a phenomenon derived from macroscopic thermodynamics under non-equilibrium conditions and, unlike a previous mathematical study (Walter et al PRL 2017, [38]), is amenable to quantitative discussion. However, we have not previously verified whether the chemophoresis quantitatively reproduces the directed motion of plasmids in a bacterial cell for the experimentally reported parameter values. Therefore, we reran the simulation with parameters that reflect the intracellular conditions and reproduced the directed motion (S7_Fig, S1_Table, newly added results) to verify it quantitatively.

(d) The appearance of directed motion depends on cell length (system size) (S7_Fig B, newly added results); since cell length monotonically increases with the cell cycle progression, the emergence of the directed movement is predicted to be cell cycle dependent.

(2) What this study can explain for the first time

As explained in #2-2 and #2-3 (1c), since our theory is derived from macroscopic thermodynamics under nonequilibrium conditions, it has **(a)** "theoretical robustness" that does not depend on individual microscopic models constructed for each molecular mechanism, and **(b)** unlike a previous mathematical study (Walter et al PRL 2017, [38]), quantitative arguments are possible. Indeed, we re-ran the simulation with parameters that reflect the intracellular conditions and reproduced the directed motion (S7_Fig, S1_Table, newly added results). Furthermore, the robustness of the theory allows its application to other systems with common reaction mechanisms (such as ATP hydrolysis). In addition, **(c)** the chemophoresis engine can be modeled as a coupled system among the reaction-diffusion equation for ParA-ATP and the equations of motion

for plasmids, allowing the application of dynamical system theory. **(d)** Plasmid size and cell length dependence has not been reported in the previous mathematical studies. Particularly, the finite size effect is parts of the novel results of the present study.

We thank you again for the clarification, which became clearer after we followed your suggestion in our discussion.

In the revised manuscript, we have added the analytical results in the zero size limit ($l_b \to 0$) (S6_Fig) and the simulation results with parameters reflecting intracellular conditions and explanations (S7_Fig, S1_Table,) and have rewritten the two main parts of the manuscript as follows:

[Page 7~8]

Plasmid size is a relevant parameter for the emergence of its directed movement

In the limit with vanishing plasmid size $l_b \to 0$, the mathematical form of the present model is reduced to our earlier model (Models). However, whether any spontaneous directed motion emerges in the limiting case or not was not discussed in our previous report [3]. In such vanishing size limit, χ and ε diverge: $\chi = kN/2l_b \to \infty$ and $\varepsilon = \mathcal{D}N/2l_b \to \infty$; nevertheless steady surfing-on-wave solutions can exist in a certain range of k, $\mathcal{D}$, and N. By using the analytical solution obtained above (Eqs S7 - S9) and by taking a large system size limit $L \to \infty$, the steady velocity in the limit is analytically obtained as (Eq S15, S1_Text): $v = \pm\sqrt{[kN(\mathcal{D}N/2+1)+2][kN(\mathcal{D}N/2+1)-2]}$. This analytical representation has a bifurcation at $k = k_c = 2/[N(\mathcal{D}N/2+1)]$: for k larger than this critical value, there exist two real-valued solutions representing the directed movement. Corresponding to Figs 2A and 2B, we obtained the phase diagram and the relationship between $|v|$ and k for $\mathcal{D} = 0.05$ and N=40, for the parameter values adopted in Figs 1 and 2 (S6_Fig). The bifurcation point k_c(=0.025) in the vanishing size limit (B of S6_Fig) is smaller than the corresponding value ($k_c \sim 0.031$) for $l_b = 0.2$ in Fig 2B, suggesting that the directed motion for a larger l_b requires a larger k, and probably N as well.

We also examined l_b dependency of the directed motion of the plasmid for the cases $N = 10, 20, 30, 40, 50$, using the analytical solution Eqs S7 - S9 (Fig 2D). There were no

solutions showing the directed movement for $N = 10$, whereas, for $N = 20, 30, 40, 50$, there exists a solution with the directed movement, whose velocity monotonously decreased with l_b, and then an inverse pitchfork bifurcation occurred at a critical value of l_b, resulting in the only solution with $|v| = 0$ (Fig 2D). Next, we numerically calculated l_b dependency of the velocity and confirmed the disappearance of the traveling wave solution with the increase in l_b (Fig 2D, black dot plots). The numerical results showed slight deviation from the analytical solution for the ranges of small and large l_b, suggesting the breakdown of the approximation $u(x) \gg K_d$. In general, the directed motion with larger l_b requires a larger k and N. This is because a plasmid (or bead) with large l_b cannot generate sufficient concentration gradient and the resultant chemophoresis force is not sufficient to realize its self-driven directed motion. Finally, these results indicate that plasmid size is a relevant parameter for the emergence of its directed movement.

[Page 8~9]

Chemophoresis engine can work in *in vivo* conditions

From the normalized equations for chemphoresis engine (Eqs 3 and 4), we here confirmed that plasmid surfing on the traveling wave emerged through a symmetry-breaking transition with the change of χ and ε (Fig 2). However, it remains unclear whether the above results can be quantitatively reproduced for the parameter values reported experimentally in bacterial cells. To quantitatively confirm that the directed motion is self-organized by the chemophoresis engine, we ran the simulations again with parameters capturing *in vivo* conditions (S1_Table), as described below.

A recent study suggests that ParB localizes around plasmids by a ParA-ATP dependent phase separation mechanism to form PCs as droplets, accompanied by an enhanced hydrolysis activity of ParA-ATP through the increase in local density of ParB within the PCs, finally leading to a successful plasmid partition [76, 77]. As a simple demonstration, we carried out the simulation by simultaneously changing the maximum rate of ParA-ATP hydrolysis $\chi = kN/2l_b$ and the strength of the chemophoresis force $\varepsilon = \mathcal{D}N/2l_b$ under the control of the local density of ParB on a PC, $\rho = N/2l_b$. We increased the cell length, which may reflect the cell cycle progression, whereas we adopted the parameters

capturing *in vivo* conditions listed in S1_Table. We changed ρ and the cell length $= L \times l$ with L as a normalized system size, scaled by $l = \sqrt{D_u \tau} = \sqrt{D_u / a} = 0.4 (\mu m)$, while ρ is a dimensionless relative density normalized by the cytoplasmic one of ParA-ATP assigned to u_0 (See also Models for normalized parameters and S1_Table for *in vivo* parameter values).

We have performed simulations for the single plasmid ($M = 1$, S7_Fig upper) and the two-plasmid ($M = 2$, S7_Fig bottom) cases using Neumann boundary condition as in Fig 1. We investigated how the directed (or pole-to-pole oscillatory) movement of plasmids emerges with the increase in ρ, by calculating time-averaged values of velocity for different cell lengths (S7_Fig A). The directed motion appeared at a $\rho = \rho_c$ for cell lengths greater than $0.8 (\mu m)$ for $M = 1$ (S7_Fig A1) and $1.6 (\mu m)$ for $M = 2$ (S7_Fig A2), demonstrating that the chemophoresis engine can recapitulate plasmid surfing even in *in vivo* conditions. Interestingly, the bifurcation points differ by different cell lengths; that is, the emergence of directed movement can be cell-length dependent. This bifurcation to the directed motion against the cell length occurs for both $M = 1$ and $M = 2$ (S7_Fig B1 and B2). In addition, the bifurcation diagram for $M = 1$ coincides with that for $M = 2$ when plotted as a function of (cell-length)$/M$ both for S7_Fig A and S7_Fig B, suggesting that controlling the inter-plasmid distance is important for the emergence of directed motion.

Finally, we have added recent references in the revised manuscript as follows:

[27] Le Gall A, Cattoni DI, Guilhas B, Mathieu-Demazière, Oudjedi L, Fiche J-B, Rech J, Abrahamsson S, Murray H, Bouet J-Y, Nollmann M. Bacterial partition complexes segregate within the volume of the nucleoid. Nat Commun 2016;7:12107.

[38] Hu L, Rech J, Bouet J-Y, Liu J. Spatial control over near-critical-point operation ensures fidelity of ParABS-mediated DNA partition. Biophys J 2021;120:3911–3924.

[43] Burkart T, Wigbers MC, Würthner L, Frey E. Control of protein-based pattern formation via guiding cues. arXiv preprint arXiv:2202.10091.

[73] Sanchez A, Cattoni DI, Walter J-C, Rech J, Parmeggiani A, Nollmann M, Bouet J-Y. Stochastic self-assembly of ParB proteins builds the bacterial DNA segregation apparatus. Cell Syst 2015;1:163.
[74] Bouet J-Y, Rech J, Egloff S, Biek DP, Lane D. Probing plasmid partition with centromere-based incompatibility. Mol Microbiol 2005;55:511.
[75] Taylor JA, Seol Y, Budhathoki J, Neuman KC, Mizuuchi K. CTP and parS coordinate ParB partition complex dynamics and ParA-ATPase activation for ParABS-mediated DNA partitioning. eLife 2021;10:e65651.
[76] Guilhas B, Walter J-C, Rech J, David G, Walliser NO, Palmeri J, Mathieu-Demaziere C, Parmeggiani A, Bouet J-Y, Le Gall A, Nollmann M. ATP-driven separation of liquid phase condensates in bacteria. Mol Cell 2020;79:293–303.
[77] Biedzinski S, Parmar B, Weber SC. Beyond equilibrium phase diagrams: enzymatic activity shakes up bacterial condensates. Mol Cell 2020;79:205–206.
