# Supplementary material for: Chemophoresis engine: A general mechanism of ATPase-driven cargo transport
Source: PLoS Comput Biol. 2022 Jul 25;18(7):e1010324. doi: 10.1371/journal.pcbi.1010324 (PMC9363008; doi:10.1371/journal.pcbi.1010324)
Supplement: Attachment — Submitted filename: ReplyToReviewers_2ndRound_PLCB_20220613.pdf [file pcbi.1010324.s015.pdf]

Point-by-point responses to comments made on the previous version of the manuscript (The original comments are italicised and shaded.)

All comments of Reviewer #1:

My referee comments have been adequately addressed in the revised manuscript. Especially, my concerns about the parameters in relation with real measurements are well addressed. Some claim of the paper is a bit hypothetical but still interesting to read. I think it is worthwhile for publication in PLOS Comp. Biol.

Thank you for your comment. We are very pleased with your positive comments. We appreciate your useful suggestions so far. Thank you very much.

All comments of Reviewer #2:

The authors addressed my comments and emphatically clarified their new findings. I'd only suggest one change: The authors claimed that "Plasmid size and cell length dependence has not been reported in the previous mathematical studies." This is not true. The new paper by Hu et al (ref #38) addressed the PC size scaling. It would make sense that the authors modify the relevant paragraphs (e.g., lines 239-268) to make connection to the findings in ref #38 and to parse out the commonality and differences.

Thank you for your comment.
Reading Ref [38] elaborately, we added in the main text the following two points in order to respond to your comment:

(1) About a dependence on partition complex (PC) size

We have added at the lines **268-269** the following sentence:

"Actually, in a previous mathematical study [38], its size relative to cell length was reported to play a different role in plasmid partition than the present result."

(2) About a dependence on cell length

We have added at the lines **302-304** the following remark:

"Such a scaling for bifurcation diagram among different values of M, as was mentioned in [38], can enable robust equi-positioning of plasmids during cell elongation [17, 67]."

Finally, we appreciate your useful suggestions so far. Thank you very much.
